# Development and evaluation of a de-identification procedure for a case register sourced from mental health electronic records

**DOI:** 10.1186/1472-6947-13-71

**Published:** 2013-07-11

**Authors:** Andrea C Fernandes, Danielle Cloete, Matthew TM Broadbent, Richard D Hayes, Chin-Kuo Chang, Richard G Jackson, Angus Roberts, Jason Tsang, Murat Soncul, Jennifer Liebscher, Robert Stewart, Felicity Callard

**Affiliations:** 1 King’s College London (Institute of Psychiatry), London, UK; 2 University of Sheffield Department of Computer Science, Sheffield, UK; 3 South London and Maudsley NHS Foundation Trust, London, UK; 4 Durham University, Durham, UK

**Keywords:** De-identification, Anonymisation, Electronic health records, Psychiatric case register, Medical health records security, Medical information database security

## Abstract

**Background:**

Electronic health records (EHRs) provide enormous potential for health research but also present data governance challenges. Ensuring de-identification is a pre-requisite for use of EHR data without prior consent. The South London and Maudsley NHS Trust (SLaM), one of the largest secondary mental healthcare providers in Europe, has developed, from its EHRs, a de-identified psychiatric case register, the Clinical Record Interactive Search (CRIS), for secondary research.

**Methods:**

We describe development, implementation and evaluation of a bespoke de-identification algorithm used to create the register. It is designed to create dictionaries using patient identifiers (PIs) entered into dedicated source fields and then identify, match and mask them (with ZZZZZ) when they appear in medical texts. We deemed this approach would be effective, given high coverage of PI in the dedicated fields and the effectiveness of the masking combined with elements of a security model. We conducted two separate performance tests i) to test performance of the algorithm in masking individual *true PIs* entered in dedicated fields and then found in text (using 500 patient notes) and ii) to compare the performance of the CRIS pattern matching algorithm with a machine learning algorithm, called the MITRE Identification Scrubber Toolkit – MIST (using 70 patient notes – 50 notes to train, 20 notes to test on). We also report any incidences of *potential breaches*, defined by occurrences of 3 or more true or apparent PIs in the same patient’s notes (and in an additional set of longitudinal notes for 50 patients); and we consider the possibility of inferring information despite de-identification.

**Results:**

True PIs were masked with 98.8% precision and 97.6% recall. As anticipated, potential PIs did appear, owing to misspellings entered within the EHRs. We found one potential breach. In a separate performance test, with a different set of notes, CRIS yielded 100% precision and 88.5% recall, while MIST yielded a 95.1% and 78.1%, respectively. We discuss how we overcome the realistic possibility – albeit of low probability – of potential breaches through implementation of the security model.

**Conclusion:**

CRIS is a de-identified psychiatric database sourced from EHRs, which protects patient anonymity and maximises data available for research. CRIS demonstrates the advantage of combining an effective de-identification algorithm with a carefully designed security model. The paper advances much needed discussion of EHR de-identification – particularly in relation to criteria to assess de-identification, and considering the contexts of de-identified research databases when assessing the risk of breaches of confidential patient information.

## Background

Electronic Health Records (EHRs) function as single, complete, integrated electronic versions of the traditional paper or part-electronic health records [1]. In 2002, an England-wide implementation, which aimed to improve patient care delivery, led to the growth of standardised electronic health record keeping systems in hospitals across England [2]. Soon after, the South London and Maudsley NHS Foundation Trust (SLaM), one of the largest mental health care providers in Europe, developed an Electronic Psychiatric Clinical Records (EPCR) system, namely the electronic Patient Journey System (ePJS) [1]. This replaced the previous system – of hard copy case notes, a community-based electronic clinical record, an inpatient patient administration system, adult mental health and addictions service administration systems – with a single, completely electronic system, where daily activities, medication, diagnosis, correspondence and any other information pertinent to patients attending this mental health care specific facility are recorded [1]. Information on ePJS can be accessed across all sites of SLaM by authorised clinical staff. ePJS has been active for around 8 years and as of October 2012, it carries records (legacy and active) for over 200 000 patients. As with many EHRs, scope for using this electronic psychiatric case register as a data source for research has been explored, opening doors for better secondary research in psychiatric health.

EPCRs have a unique place in research [3]. Information recorded in psychiatric registers is relatively more sensitive compared with other clinical registers [4,5] and includes material that contains the potential for stigmatization and discrimination. Studies indicate that when consent has been sought to use a person’s health data for research, refusals tend to be most common if the research involves matters of sexual or mental health [4,6]. A study sampling 15,997 general practice outpatients in Minnesota investigating the impact of requiring patient authorisation on research using medical records encouragingly found low refusal rates overall (576 patients refused authorisation); of those who refused, the greatest proportion was among those seen for mental health reasons (8.5%) [6]. Another study by Powell et al. of 50 consecutive primary care attenders found that the most common items that patients did not wish to share were related to mental health [7]. This could give rise to biased samples when conducting mental-health-based studies. In addition, the findings indicate that if the sample were entirely patients in a mental health setting, a higher number would refuse sharing information for research, potentially leading to low numbers and reduced generalisability [6,7]. To avoid such drawbacks and to encourage patient trust in use of patient data, de-identification might offer a better route to use medical records, in particular psychiatric records, for research [4]. In many jurisdictions, the requirements to use any EHRs (including electronic psychiatric health records) for research purposes usually stipulate a “consent or anonymise” approach. Anonymisation requires replacing, removing or *de-identifying* information directly related to the patient, referred to here as Patient Identifiers (PIs) [8], so as to allow these medical records to be harnessed for research purposes. If the patient has given consent to use his or her records for research, then there is no need to de-identify. On the other hand, an anonymised database is one where the original database has been stripped of PIs and where the identifier number assigned to each individual has been replaced with an anonymised identifier that is not linked in any way to the original dataset.

A pseudonymised database is the same as the anonymised database, except the original identifiers are in a securely linked table, typically with a trusted third party.

A de-identified database is a database that typically replaces or removes all PIs, as defined by specific national regulations. While it is relatively straightforward to mask *structured* fields containing identifying information, this is more difficult for *free text* (or unstructured) fields in EHR-sourced datasets, although it is here that many of the most valuable data are contained, particularly for mental healthcare.

In the United States, the Health Insurance Portability and Accountability Act (HIPAA) specifies 18 PIs that need to be removed or replaced to de-identify medical records to be used in research; these include patient and non-patient addresses, as well as all dates (except years) relating to an individual [9,10]. The U.K., on the other hand, provides broader guidance on the types of identifiers that need to be de-identified, acknowledging that a “truly anonymous dataset is unlikely to be useful for much research” [8] and that appropriate de-identification has to be designed based on the *context* of usage. Generally, in the U.K., the concept of de-identifying medical records means: i) replacing “key identifiable information” or key patient identifiers (Table 1) [8,11]; ii) partially removing strong PIs (e.g. date of birth or post codes) so as to make them weak PIs (e.g. provide month and year of birth and the first half of the post code) while maintaining research value [8]; and iii) aggregating rare characteristics [8]. Each National Health Service (NHS) organisation in the U.K. is entrusted with a “Caldicott Guardian” and an accompanying committee (which involves clinical and service user representation) [12]. The overriding duty of the Caldicott Guardian and the Caldicott Committee is to ensure that patients’ confidentiality is properly ensured in any context within the Trust. Any researcher attempting to use medical records for research must consult and work with the Caldicott Guardian and committee to agree on an appropriately de-identified database, keeping in mind the implemented national regulations on patient data confidentiality, as well as the technical and management challenges of creating a research database [8].

**Table 1:** List of key Patient Identifiers (PIs) specified to be de-identified in the U.K. to create a de-identified database as stipulated by the Caldicott Code on Confidentiality

	
**What are patient identifiers?**	First Name
	Middle Name
	Last Name
	Current and Old Address Line 1
	Current and Old Address Line 2
	Current and Old Address Post code
	Current and Old Telephone Numbers
	Current and Old Email addresses
	Date of birth
	National Health Service (NHS) Identification (ID) numbers*
	Hospital specific ID numbers
	Rare or unique characteristics
	Aliases/Nicknames

*NHS numbers are assigned to every resident of the U.K.

These are all personal to the patient. There is no obligation to de-identify all non-patient information such as clinical staff names.

SLaM undertook the initiative to build the Clinical Record Interactive Search (CRIS) in 2007, and in 2008 CRIS received research ethics approval for use as an interactive, de-identified database for secondary research in mental health. The need for robust de-identification of psychiatric case registers is particularly acute: both to enable representative, reliable and valid evidence-based research in psychiatric research, and to protect patient identity and consolidate patient trust vis-à-vis use of intimate data. CRIS has now been active for over 4 years and is used by clinical staff and researchers to conduct audits of services and research projects involving secondary data collection. CRIS has contributed to research on Alzheimer’s disease, severe mental illness and its associations with mortality, early-stage psychosis and other more rare disorders or syndromes [13-20]. The search system enables specification of criteria that define the cohort of interest (for example, patients with a given diagnosis or all those whose records contain a key word or phrase within a given time period) and then brings back any variables of interest (for example, a structured field such as ethnicity, or a free text field such as those used for case notes). Based on these parameters, the CRIS application will bring back a database of cases that meet the search criteria (rows) with the requested output variables for each case (columns).

The development of CRIS as well as descriptive data have been described in detail [1] elsewhere. We focus here on the development, implementation and evaluation of the de-identification design for ePJS (to create CRIS), which emerged through a series of discussions and meetings that involved the Caldicott Committee, as well as a Stakeholder Committee (which incorporated service user representation and leadership, as well as additional service user consultation). The main criteria according to which a de-identification algorithm was designed comprised: i) the protection of patient identity (including the sensitivities that come with this database being a mental health care database – e.g. unique, potentially stigmatizing, mental health-related events described in the text), and ii) the maximisation of research value (by maintaining the characteristics of the psychiatric database and avoiding over-de-identification).

### CRIS – a combination of the de-identification algorithm and security model

The algorithm (described in detail in the Methods section) that was approved by the ethics board still had its share of limitations, as do many other de-identification algorithms [21]. We summarise these here, prior to providing the specifics of the algorithm, so as to provide readers with the broader context surrounding the development of CRIS, as well as to clarify some of the decisions that were made surrounding de-identification. In brief, the algorithm does not identify PIs occurring in the CRIS text that are not entered in the source system (*un-entered PIs*). This is because, as we describe later on, the dictionary that our algorithm uses is populated using data filled in dedicated PI fields in the source system. Hence, any information not entered in these fields will not be included in our dictionary and will not be recognised when it occurs in free text. Additionally, misspelt PIs in the source system are not identified as PIs in CRIS text and hence are not de-identified. There are two points to note here.

Firstly, it is possible for misspelt PIs and un-entered PIs appearing in the CRIS text to compromise patient anonymity. These issues were discussed before the launch of the CRIS system by the Caldicott and CRIS Stakeholder committees. A culmination of validation tests, discussions and trials led to a consensus view that instances of misspellings, nicknames, and un-entered pieces of information occurring in isolation would not pose a threat to identity in the absence of any other correct PI (for example, the appearance of an incorrect date of birth with no other information related to the patient). Secondly, the dependency of our dictionary on data populated in source may come across as a limitation; however, the PI fields (in the source system) that contribute to our dictionary are either mandatory fields to populate (in the source system) or are fields that are highly populated. The current total population of SLaM is greater than 200 000 (these include legacy and active patients); of these, all patients have a populated first and last name field, as it is mandatory. Almost all of the patients, 99.7%, have a valid date of birth filled in, and 98.5% of them have at least one address entered. These high coverage figures provided reassurance that we could develop our dictionary from these data. The other reason why we invested trust in creating a dictionary from source fields is because the content of the record is informed by *clinical utility* (i.e. we can make reasonably safe assumptions that if data are not entered in the relevant PI forms, it is because they are not known and therefore will not be included in other parts of the records – such as daily notes or correspondence notes). If this information comes to be known later on, it would be the role of the clinical staff to enter the information in the relevant source forms. (To test this assumption, we did a search on records that did *not* have a date of birth filled in and searched in their correspondence notes for references to “date of birth”. Out of 517 patients who do not have a valid date of birth, 10 records were returned with a reference to “date of birth” in their correspondence notes. Three were never admitted (though were referred) to the hospital in the first place (and hence did not have the date of birth entered in the source field). The remaining 7 records did have reference to “date of birth”, and these were fed back to the records administration team to enter proper date of birth in the source records to ensure masking on the CRIS system.) Based on this assumption as well as the high coverage of the strong patient identifier fields on the source system, we determined that building the CRIS PI dictionary using the source fields would suffice to mask any mention of PIs in the free or structured text.

This being said, realistic possibilities of inference of patient identity from misspellings, un-entered PIs, nicknames, unique data or any other potential to recognise individuals cannot be ignored for medical data. With this in mind, the CRIS Stakeholder and Caldicott committees established, in line with U.K. legislation on data de-identification for research, a rigorous security model in which to enmesh the CRIS de-identified dataset (described in detail in the Methods section). Together with the technical component, this security model plays an equally important role in establishing a robustly de-identified psychiatric research database. In essence, the security model was designed to deal with the realities of de-identifying databases, through acknowledging that all datasets will have inaccuracies which will compromise performance of the de-identification algorithm [22]. In the Methods section we explain the technicalities of the algorithm, the process for de-identifying records, the security model and the evaluation study. To the best of our knowledge, there is no literature thus far describing the de-identification of psychiatric case registers [10,23].

## Methods

### CRIS de-identification process and algorithm

**Figure 1:**
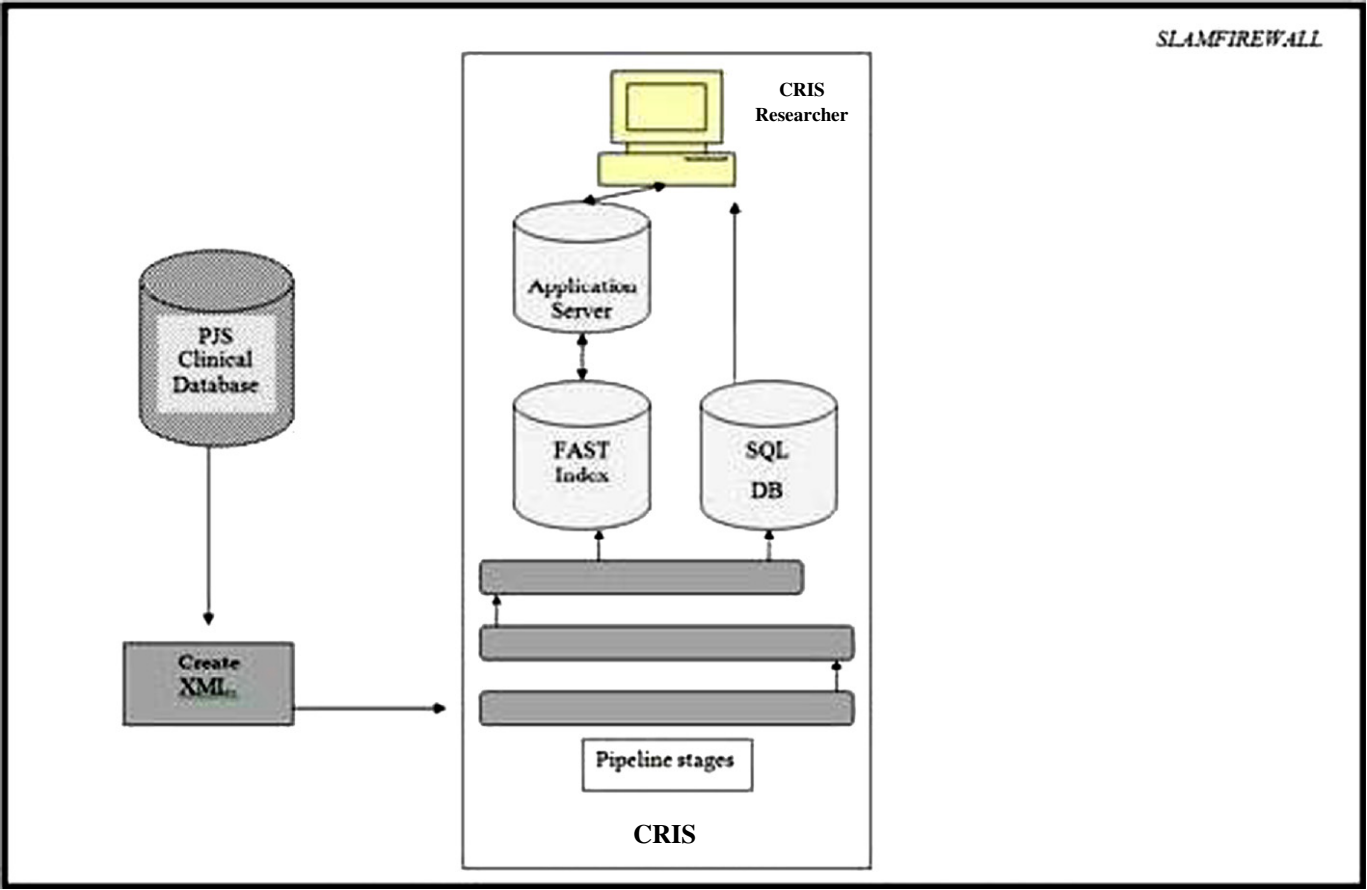
Diagrammatic description of converting source medical records (Electronic Patient Journey System [ePJS]) to Clinical Record Interactive Search (CRIS).

**Figure 2:**
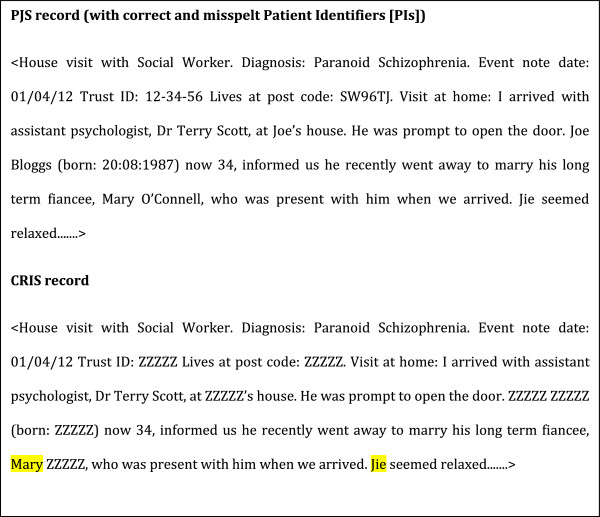
**Source Electronic Patient Journey System (ePJS) record input and CRIS output.** Note that in this example we are using the dictionary from Table 2. “Jie” and “Mary” have not been masked because of being a typographical error and un-entered PI, respectively. All details in this record are fictitious: any resemblance to real persons is entirely coincidental. Also note that there are no legal requirements to de-identify names of clinical staff such as “Terry Scott”, the fictitious assistant psychologist, whose name therefore appears in full in the CRIS record.

To create a data store of de-identified ePJS records, a step-by-step process was followed to mask all PIs. Information populated on the html front-end of ePJS (Figure 1) is processed and stored in a secure Structured Query Language (SQL) database, which is updated as and when new information is entered (on the front end). To populate the CRIS search engine (Microsoft FAST™ Enterprise Search Installation - the contents of which are copyrighted and not disclosed here) with ePJS source data, the data need to be converted into Extensible Mark-up Language (XML). This is achieved by extracting all content from a replicated SQL Server using a custom program called SQL extractor. The SQL extractor code creates one XML document per patient and stores all the details relevant to the patient in this document, maintaining the same hierarchical structure in which they are recorded on ePJS. Once the SQL Extractor has finished extracting the source SQL data into the required XML format, the patients’ records of various sizes are stored on disk before being passed to FAST for ingesting. The FAST process ultimately results in the production of a searchable index. During the production of the index, the XML records are modified using a transformation pipeline written in Python that is accessed from within FAST. Different stages during the transformation pipeline are responsible for the de-identifying of the patient record. The de-identification process is handled by two pipeline stages which take in different configurations, so that the code can determine not only what needs to be removed from the XML but where in the XML the PI content can be found. Each XML record is then passed through the pipeline stages for processing. We have outlined the process below:

i) A list of all fields that contain PI information – dedicated source fields. Along with the field name, a field type is defined so that the code is aware of how the values of the given field should be treated when being added to the cleaning dictionary. For example, if the field type defines it to contain information on the name of the patient, the information will be added to the cleaning dictionary *and* in addition this field will be stripped from CRIS completely so as not to appear at all in CRIS. If, on the other hand, the field contains postcode information, the code will add the postcode to the dictionary *and* in addition, will truncate this field so that only the first half (3 or 4 letters) of the postcode appears in CRIS (this is to mask patient identity and maintain research value by retaining some geographical information). Unlike many other de-identification algorithms, CRIS does not derive its dictionary from population registries [23]. Instead, the dictionary is populated with terms derived from the patient forms in the source EHR (Table 2). This method has been used in other de-identification systems [24,25]; however many have been supplemented with tools to recognise misspelt names such as the name nearness matching [23,25,26]. A CRIS dictionary (also known as the cleaning list) hence will be different for each patient. Information from patients’ first names, last names, contact numbers, key person contacts, addresses and alias/former name fields are extracted from the source, stripped of delimiters (such as apostrophes and hyphens in names) and added to the dictionary.

ii) A list of *XML tags or scopes*. To save processing time, the de-identification algorithm is not run across the whole of the XML. Instead, areas – called *XML tags* or *XML scopes* - are flagged for the de-identification algorithm and they determine where the algorithm should run. These essentially are all “free text” fields (e.g. the case note and correspondence fields, where almost 60% of all data on CRIS are recorded), core patient information forms and summary title fields. Typically, all fields in which there is a possibility of mentioning a PI have been assigned as XML tags. A list of these is presented in Table 3.

iii) A masking string to indicate what the removed words should be replaced with. Words are replaced with ZZZZZ for patient identifiers and with QQQQQ for relative or close contact identifiers (see Figure 2). We chose not to replace each type of PI with a generic word describing the type of PI (e.g. FIRST NAME, ADDRESS LINE, DATE OF BIRTH) as there is usually sufficient contextual information from which it is possible to deduce what the type of PI could be.

**Table 2:** An example of the CRIS dictionary

**ePJS Source fields**	
First name	Joe
**Middle name	(blank)
Second name	Bloggs
*Date of birth	20/08/1987
Trust ID	12–34–56
Post Code	SW9 6TJ
**Nick Name	(blank)
**Key Contact First Name	(blank)
Key Contact Last Name	O’Connell
**CRIS PI Dictionary list derived from the source field above**	
Joe	
Bloggs	
20/08/1987	
20/08/’87	
20–08–1987	
20–08–87	
20.08.1987	
20.08.87	
20.8.87	
20^th^ Aug 1987	
20^th^ Aug ‘87	
20^th^ of August 1987	
20^th^ of Aug 1987	
12–34–56	
123456	
12 34 56	
SW9 6TJ	
SW96TJ	
Connell	

*There would be many more options of recording the date of birth; a non-exhaustive list is provided here.

**The cells show no information was entered in these fields and hence are not included in the CRIS PI dictionary. Note that these fields are patient identifier fields that sometimes do not get filled in the source.

**Table 3:** List of XML scopes or XML tags, through which the de-identification algorithm is run

	
**All free text fields**	Summary texts, Event notes, Correspondence notes, Ward Progress Notes, etc.
**All structured fields that would be populated, in the source system, with strong PIs are completely removed on CRIS**	NHS number field, Trust specific ID field, First Name, Middle Name, Last Name, Telephone Number, etc.
**All structured fields that would be populated, in the source system, with strong PIs that are also valuable for research, are converted into weak identifiers**	Date of Birth, Post Code, Ethnicity, etc.

iv) Address aliases. A list of known variations in addresses (e.g. road/rd, street/st,) helps the cleaning processes find instances of addresses that may not have been entered identically to what was placed in the address fields. Address aliases are a particular focus, because any details appearing in the CRIS text regarding current patient address pose a stronger threat to anonymity compared to any other PI.

With the abovementioned information, the python code uses the configured fields to build up the cleaning dictionary. As the dictionary is being built, heuristics are used to accommodate for different PI formats (or patterns). This is an important feature that helps CRIS recognise all forms of PIs (or patterns) mentioned in the text, particularly through accommodating the variety of formats in which a PI can be recorded in free-text fields. For example, a mobile telephone number can be written in a standard format (00000 – 000 – 000) or can be written out as one number with no spaces (00000000000). Tables 4, 5 and 6 are examples of the heuristics written for names, date of births and postcodes, respectively. All the other heuristics written to capture word dates, phone numbers, NHS numbers and addresses are presented in Table 7.

**Table 4:** Heuristics to identify names, with entirely fictitious examples, as they would appear in the source record and CRIS output

	**Algorithm to identify names**					
	**<beginning > <*name term*** **> <*optional_name_delimiters*** **> <*optional_s*** **> <end >**					
**Source record**	**<beginning>**	**<*name term *>**	**<*optional_name_delimiters *>**	**<*optional_s *>**	**<end>**	**CRIS de-identified output**
…replaced. Mark will also be able…	.(space)	Mark	None	None	(space)	…replaced. ZZZZZ will also be able…
…knowing Mark’s diagnosis…	(space)	Mark	‘	S	(space)	…knowing ZZZZZ diagnosis…
…7)Mark is compliant…	)	Mark	None	None	(space)	…7)ZZZZZ is compliant…
…OMark is compliant…	No beginning identified	None	None	None	None	…OMark is compliant…
…was awarded 9 mark out of 30 in…	(space)	mark	None	None	(space)	…was awarded 9 ZZZZZ out of 30 in…
…Nurse informed Mark. Earlier…	(space)	Mark	None	None	.	…Nurse informed ZZZZZ. Earlier…
…Marik will be attending…	(space)	None identified due to misspelling	None	None	None	…Marik will be attending…
…O’Mark is at the…	O’	Mark	None	None	(space)	…ZZZZZ is at the…
…his father, John, was also present…	, (space)	John	,	None	(space)	…his father, QQQQQ, was also present…

**Table 5:** Heuristics to identify date of birth, with entirely ficitious examples, as they would appear in source records and CRIS output

	**Algorithm to identify date of birth (Number date) <beginning > <day|month|year > <one_date_delimiter > <day|month|year > <one_date_delimiter > <day|month|year > <end>**							
**Source record**	**<beginning>**	**<day|month| year>**	**<date_delimiter>**	**< day|month|year>**	**<date_delimiter>**	**<day|month|year>**	**<end>**	**CRIS output**
Dob: 01/01/2001	:	01	/	01	/	2001	(space)	Dob: ZZZZZ
1^st^ of January 2001	(Space)	1^st^	(space) of (space)	January	(space)	2001	(space)	ZZZZZ
…born in Jan 1^st^ 01…	(space)	Jan	(space)	1^st^	(space)	01	(space)	…born in ZZZZZ…
…01-01-’01…	(space)	01	-	01	-‘	01	(space)	…ZZZZZ…
…01 Jan 2001	(space)	01	(space)	Jan	(space)	2001	(space)	…ZZZZZ…
Dob: 01//01/2001	:	01	/	None identified owing to typographical error in the source record	None	None	None	Dob: 01//01/2001

**Table 6:** Heuristics to identify post codes, with entirely fictitious examples, as they would appear in source records and CRIS output

	**Algorithm to identify post codes < postcode>::= source postcode**	
**Source record**	**Source postcode**	**CRIS output**
He lives at EN1 5SR	EN1 5SR	He lives at ZZZZZ
Lives at EN1. No…	None	Lives at EN1. No…
Lives at EN1 S5R	None identified owing to typographic error	Lives at EN1 S5R

**Table 7:** Algorithms to de-identify date of birth (word form), phone numbers, NHS identification numbers and addresses

	
	Algorithm to identify date of birth (Word date)
**<word_date>**	::= < beginning > <day > <spaces > <day_suffix > <spaces > <optional_word_delimiter > <spaces > <word_month > <spaces
	> < optional_comma > <spaces > <year > <end > | < beginning > <word_month > <spaces > <optional_month_delimiter > <spaces > <day > <spaces > <day_suffix > <spaces > <optional_comma > <spaces > <year > <end>
	
	Algorithm to identify phone numbers
**<phone_number>**	::=<optional_open_bracket > <first 5 digits > <optional_close_bracket > <space > <digits 6-8 > <space > <digits 9-11 > |
	<optional_open_bracket > <first 3 digits > <optional_close_bracket > <space > <digits 4-7 > <space > <digits 8-11 > |
	<optional_open_bracket > <first 4 digits > <optional_close_bracket > <space > <digits 5-7 > <space > <digits 8-11>
	
**<nhsnumber>**	Algorithm to identify NHS Identification numbers ::= < numeral^1^ > <number_delimiters > <numeral^2^ > <number_delimiters > … < numeral^n^>
	
	Algorithm to identify addresses
**< address>**	::= < address_term^1^ > | < address_term^2^ > …. <address_term^n^>

Once the dictionary is complete, the python code reads in the XML tags and uses a regular expression to determine terms and tokens within the XML tags that need to be removed from each record. This is based on a set of rules for each token/term to determine where both the *start* and *end of the token* can be found. The *start of a token* is defined as i) one where the immediate prefix does not contain letters or numbers, ii) where the prefix is the start of a sentence or iii) any word which start as a new line (i.e. there is no prefix). The *end of a token* is defined as i) one whose suffix does not contain letters or numbers or ii) whose suffix is the end of a sentence (indicated by full-stops, commas, colons, semi-colons and hyphens). Once a token/term has been identified, it is compared against the cleaning dictionary, and when a match is found the resulting token is replaced with the required masking string. A python code implements the de-identification algorithm to create the dictionary and then identify, match and replace PIs in the text. This code can be configured to add fields or tags without having to change the underlying code base.

### The CRIS security model

The CRIS Oversight Committee (which evolved from the Stakeholder Committee, after CRIS received research ethics approval as a de-identified database) comprises the central governance entity overseeing security. Access to CRIS is application-based. Potential users submit an application to the CRIS Oversight Committee, in which they are asked to describe their project and the variables of interest. The committee, chaired by a mental health service user, also includes a child and adolescent mental health clinical representative, a representative of the Trust’s Caldicott Guardian, a Research Ethics representative, the CRIS academic project lead and the CRIS project manager. Potential applications looking to conduct audit of clinical services using CRIS need to gain approval from the relevant audit committee (within SLaM) before applying to use CRIS. Likewise, research project applicants need a senior university or NHS affiliated supervisor attached to and taking responsibility for the project and applicant before applying to use CRIS. Each applicant must have a formal affiliation in the form of an honorary or substantive contract with the hospital or the university before applying to access CRIS. These formally bind the applicant to the NHS duty of confidentiality when dealing with patient data (including de-identified patient data) [27].

Upon submission, the Oversight Committee determines whether a project is deemed suitable to access the CRIS database. “Suitability” is ascertained by verifying the need for the project, the scientific robustness of the application, and any patient confidentiality concerns to which the project may give rise. Any projects with the potential to identify patients, such as those investigating rare disorders or outcomes, are carefully discussed with the researcher and their supervisor and, where possible, alternatives provided (for example, the applicant is encouraged to obtain patient consent).

**Figure 3:**
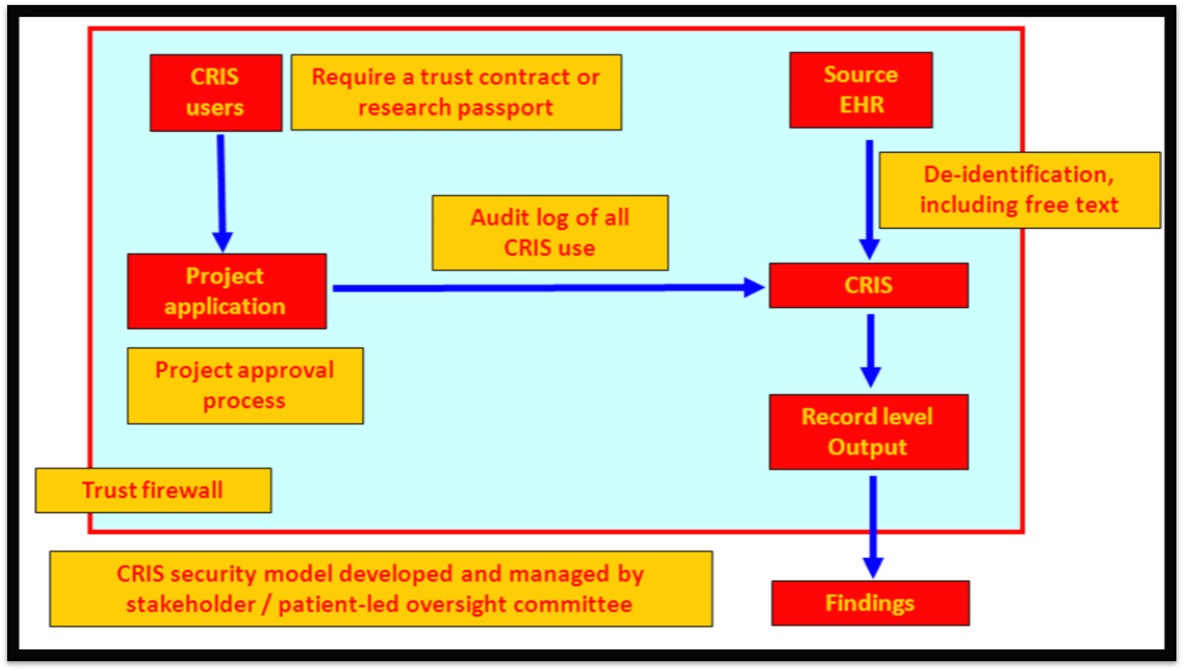
CRIS security model.

If researchers receive approval to use the CRIS system for the submitted project, they are permitted to access CRIS only within the SLaM security firewall and must follow a set of rules which facilitate responsible handling of data and uphold duties of confidentiality. All projects are audited weekly to ensure searches are being carried out within the remit of the submitted and approved project. Approval to use CRIS can be withdrawn in cases where inappropriate searches have been made in violation of the terms of the approved project. These procedures focus on close regulation of access to CRIS, as well as close monitoring of use of CRIS (Figure 3). The researcher must commit to ensuring that s/he will uphold the NHS duty of confidentiality when handling the data and adhere to the guidelines set out by CRIS (including not carrying data out of the Trust firewall for any purpose). In this way, the security model endeavours significantly to mould the researcher’s intentions – and hence behaviour – when encountering the data, so as to minimize any threats posed to patient anonymity identified above.

### Evaluation of CRIS

We evaluated the CRIS de-identification algorithm on performance in two ways. Firstly, by presenting precision and recall; also by separately comparing its performance to a machine learning de-identification tool – the MITRE Identification Scrubber Toolkit (MIST) – developed by the MITRE Corporation [28] to ascertain CRIS’ performance relative to this open source algorithm [28]. Secondly, we evaluated the potential to infer information (acknowledging that this is a standard limitation of all de-identified research databases). The CRIS evaluation study was conducted in April 2012 and hence the same set was not used during the development of the CRIS system. The methods used to evaluate each above-mentioned aspect are discussed below:

(i) Performance testing – precision and recall

To test performance, we extracted a random selection of 500 patients’ notes (events and correspondence) from the CRIS system to obtain recall (the number of PIs the algorithm de-identified out of all the PIs in the given text) and precision (the proportion of PIs de-identified that were correctly de-identified) for the algorithm. We specifically looked for instances of PI that were entered in the dedicated source fields and still found in the CRIS text – *true PIs*. It is important to note that misspellings and PIs not entered in the source patient information forms but mentioned in the free text (*un-entered PIs*) were excluded in the first part of the evaluation study, as the algorithm had not been designed to capture these. Each note was compared with its corresponding note in the source system to ascertain what should be de-identified.

To test CRIS performance with the MIST system, we decided to train MIST with a set of 50 day-to-day events notes and, we trained it to de-identify First Names (FN) and Last Names (LNs) only for ease of analysing data and training the model as accurately as possible (in depth details of how MIST works is published in Aberdeen et al., 2010) [28]. We present separate recall and precision rates for MIST and CRIS in Table 8.

(ii) Potential for breaches and inferring information

**Table 8:** Precision and recall rates from the machine learning approach and CRIS’ pattern matching approach

**Types of PI**	**MIST performance**	**CRIS performance**
**Total Number of Notes scanned**	20	20
**Total number of PI instances**	191	191
**Number of PIs correctly identified and masked (True Positives)**	154	169
**Number of PIs that should have been masked (False Negatives)**	43	22
**Number of instances masked that should not have been masked (False Positives)**	8	0
***Precision***	*95.1%*	*100%*
***Recall***	*78.1%*	*88.5%*

To test for *potential* breaches, we manually scanned each note (500) for a potential breach. Currently, there is no set precedent for how many combinations of PIs (correct or incorrect) need to appear to constitute a potential breach. The CRIS Stakeholder and Caldicott committees agreed by consensus that a breach would be constituted by 3 or more correct PIs, or 3 or more misspelt but strong potential PIs, either in a single note or in a series of notes for a single individual. Additionally, we randomly selected 50 patients and scanned through their events notes longitudinally. We looked at an average of 20 sequential notes for each of the 50 patients. In doing so, we mimicked the way in which the CRIS system is currently being used by users (i.e. usually projects require scanning of more than one note for a single patient to obtain data).

We provide in the Results and Discussion section a largely narrative presentation of the possibility to infer information from the data, as CRIS presents de-identified data at patient level, and discuss the role of the security model to protect the de-identified data.

## Results

(i) Performance testing – precision and recall

Our evaluation study of 500 event and correspondence notes yielded 97.6% recall and 98.8% precision rates (Table 9). In other words, the algorithm was successful in recognising and masking correctly recorded PIs in CRIS text and the various ways in which they can be recorded in free-text. These high precision and recall in our evaluation study is a cumulative result of the technical changes made to the CRIS de-identification algorithm from regular, smaller-scale, informal tests of the algorithm conducted throughout the 3 years. (For example, one of the results from the earlier tests showed that the algorithm was not recognising PIs in different formats. The algorithm was subsequently amended to take this into account.) (Of note, CRIS also de-identifies patient relative or close contact information to increase patient anonymity, although there is no legal stipulation to do so. We do not refer to or discuss patient relative/close contact de-identification further in this paper in order to maintain simplicity. In essence, rules mentioned here are applicable to patient relative or close contact identifiers as well.)

**Table 9:** Precision and recall from the CRIS performance test

**Types of PI**	**Frequency**
**Total Number of Notes scanned**	500
**Total number of PI instances**	3603
**Number of PIs correctly identified and masked**	3573
**Number of PIs that should have been masked**	89*
**Number of instances masked that should not have been masked**	30
***Precision***	*98.8%*
***Recall***	*97.6%*

*See Table 10.

As noted earlier, during the development and post-launch of CRIS it was acknowledged that this de-identification design is limited in that in cannot de-identify misspelt PIs and *un-entered PIs*; these would still appear in the CRIS text. Within the 500 notes, we found 89 potential instances of PI: 63 instances of misspellings and 26 instances of un-entered PIs in the source. These, as expected, were not de-identified, and could be counted as potential PIs (Table 10). ‘Potential’ here implies that the source PI *might* be guessed or inferred: it does not imply an instance comprising an actual, recorded PI. (An example of a ‘potential’ PI would be a nickname that is not entered in the relevant source (Alias) forms, is hence not included in the CRIS PI dictionary, and hence will not be masked in CRIS text).

**Table 10:** Instances of ‘potential’ PIs: none of which is a breach as none occurred in isolation

	**Number of instances**	**Reasons**
**Number of ‘potential’ PI instances in the notes (within 500 notes)**	**89**	**70.9% due to misspellings; 20.9% due to PIs being un-entered in source**
**Type of Potential PIs**		
**Patient Nickname**	20	Un-entered PI
**Patient First Name**	18	Un-entered PI or misspellings
**Old Address Post Code**	14	Un-entered PI or misspellings
**Patient Date of Birth**	13	Un-entered PI or misspellings
**Patient Contact Number**	2	Un-entered PI
**Patient Last Name**	10	Un-entered PI or misspellings
**Old Address Line 1**	5	Un-entered PI or misspellings
**Patient Middle Name**	4	Un-entered PI
**Old Address Line 2**	2	Un-entered PI or misspellings
**Old Address Line 3**	1	Un-entered PI or misspellings

In our comparison study with a machine learning approach, the CRIS pattern matching based precision and recall rates fared better (Table 8) – precision 100% (CRIS) versus 95.1% (MIST) and recall 88.5% versus 78.1%, respectively. The results suggest that while a machine learning approach has the ability to de-identify misspellings or nicknames, it also has increased scope to de-identify clinical staff names and generic terms. Having said this, the machine learning algorithm was trained only after a single run and Aberdeen et al.’s paper suggests training its algorithm after several test runs [28]. Our study also showed that CRIS precision and recall on its own are high enough and with potential for incorporating an approach to mask nicknames and misspellings. We acknowledge that a machine learning approach may have performed better had we performed a second or third run; and that a combination of a pattern matching together with a machine learning approach may accomplish the masking of misspellings or nicknames as well. However this was a limitation that we had already thought of and addressed (see ‘*CRIS – a combination of the de-identification algorithm and security model’* in the Background section) during the development of CRIS. We maintain that machine learning and pattern matching have their share of advantages and disadvantages [28] and have assurance that for the purposes of CRIS use, the de-identification algorithm performs exceptionally well (Tables 8, 9, and 10) and is made further robust with our tight security model in place.

(ii) Potential for breaches and inferring information

Only 1 patient note out of 500 patient notes generated multiple appearances of potential PI material that fitted our definition of a breach (see Methods section) (Table 11). Out of the series of 50 patients’ notes manually scanned for PIs, we found, encouragingly, no notes with three or more instances of potential PIs (i.e. no breaches were found). While those instances that were found could potentially pose a threat to anonymity we argue that the intention of the researcher (cf. a researcher actively wishing to re-identify with a researcher who is attentive to the threat of identification and adhering to the duty of confidentiality) who comes across this information plays a key role in determining whether the information poses a threat to patient anonymity. Moreover, El Emam et al. emphasise that the *verification* of identity is important [22] because otherwise we cannot be sure whether the information is indeed a PI. El Emam et al. point out three reasons for verification: first, the potential PI could match another patient and hence is not specific to the patient; second, the information could be incorrect (as with our 3 misspelt pieces of information relating to patient 1, in Table 11); and third, there is no way of finding out if the information is true if the researcher (or an adversary, if s/he is actually intending to seek out identifiable information) cannot verify the information for its *correctness.* Finally, the data could be old and outdated, hence making the information potentially redundant [22].

**Table 11:** Instance of a potential breach: 3 or more PIs appearing for a single patient

***Patient***	***Type of PI***	***Year of document***	***Reason for appearance on CRIS***	***Confidence in re-identification***
1	Patient third line of address	2006	Misspellings	Low: Outdated information, and cannot be verified, and incorrect spellings [22]
	Patient post code			
	Patient last name			

It is important to emphasize that completely de-identified records and incorrect or un-verifiable information *in context* may bring about opportunities for inference breaches [29]. In fact, after scanning through the sequential notes, we did find one instance of a patient’s prison contact phone number, prison reference number and his misspelt last name. Though these do not constitute our definition of a breach (Table 11), we cannot deny that these instances of information that appear on CRIS – owing to not being entered in the source system, being misspelt or because we do not identify them in the algorithm as being PIs – may comprise data from which we can infer patient information (Table 12).

**Table 12:** Potential instances that could lead to inferring patient information

***Patient***	***Type of PI***	***Year of document***	***Number of sequential records read to obtain this information***	***Reason for appearance on CRIS***	***Confidence in re-identification***
2	Patient prison reference number	2012	12	No rule to de-identify prison reference numbers or prison contact information	Low: cannot be verified, and misspellings [22]
	Prison contact phone number			Typographical error in patient’s last name	
	Patient last name				

In addition, data on CRIS are not aggregated and information is provided at de-identified patient level. Opting not to aggregate data comes with its share of advantages and disadvantages. The advantages include avoiding considerable information loss (distortion of the original information after de-identification) [30], which makes the de-identified research database more valuable (i.e. we generalise neither age, nor date references) [8]. The major disadvantage lies in the potential to identify a patient through combinations of contextual data, even when all PIs are masked and there are no incorrect PIs. Mental health is, once again, a particularly sensitive domain in this regard: media reporting of high-profile events in which a person’s mental health status is assumed to play a role can mean that a small number of patients could be at risk of de-anonymisation by virtue of researchers recognizing contextual details in a patient’s de-identified record. The security model, as mentioned earlier, is designed to compensate for these limitations: it is intended to protect the integrity of the data as much as possible without compromising their research value.

## Discussion

### Other approaches to de-identification

To conclude our evaluation of the CRIS de-identification algorithm, we would like to point out that the CRIS de-identification algorithm was not designed to compete with other algorithms, but rather to provide a novel means of de-identifying a database in a bespoke way for psychiatric research that is likely to rely particularly on information from free text fields.

The CRIS de-identification algorithm is not designed to distinguish ambiguous names from real names; for example, adjectives or nouns that could also be names would be de-identified (e.g. the word ‘mark’ would be de-identified alongside the name ‘Mark’). However, our evaluation study did not record any de-identification of generic terms, suggesting that the probability of this over-de-identification is low. There are means to curb this if necessary: El Emam et al. describes a version of the k-anonymity algorithm (an algorithm that is commonly used to define criteria to anonymise databases), where the amount of information loss is “calculated” based on generalisation and suppression values and the optimum replacement combination is chosen to reduce information loss as much as possible [30].

Several competitive evaluation tasks have looked at de-identification, including Informatics for Integrating Biology and the Bedside (i2b2) challenges, which involve the release of fully de-identified notes from a research patient data repository so as to enhance the ability of natural language processing (NLP) tools to extract fine-grained information from clinical records [31]. These evaluation tasks are not analogous to the evaluation we describe here, for several reasons: first, the i2b2 datasets do not include the structured data used by our algorithm and which are usually available for real-life de-identification tasks; second, the competitive tasks do not consider context of use (in comparison to the CRIS context, which includes the existence of a security model and regulated access to the CRIS system); third, these competitive tasks have been applied to registers other than those used in psychiatric research [21].

Converting the SLaM psychiatric case register into a research database has drawbacks in common with other case registers, because the data are not collected primarily for research purposes. The recording of clinical notes is noisy and non-standardised, and inevitably contains typographical errors. Nonetheless, the potential research value of such information is huge [32,33]. A systematic review by El Emam et al. could not show enough evidence that identity could be inferred from de-identified databases, but highlighted claims that data could be re-identified with “relative ease” [22]. One characteristic of anonymised or de-identified registers may be that it is virtually impossible to replace all PIs, aggregate all unique information and maintain the research value of the database [8]. Data custodians and researchers may therefore need to acknowledge that this is an inevitable constraint of these kinds of data.

## Conclusion

The growing realization of the extent and depth of data found within EHRs means that both health researchers and policy makers are showing increasing interest in extracting information from textual documents within EHRs for secondary purposes [23]. There is a clear need for more comparative research on the development and evaluation of de-identification algorithms that are appropriate for use within different kinds of legislative frameworks and in relation to the demands of different kinds of health systems. There is also a need for ongoing consideration of which criteria – and which PIs – to use when assessing the effectiveness of de-identification strategies, as well of *how* and *by whom* de-identified health research databases are used in order appropriately to assess the risk of breaches of confidential patient information. We believe that our paper responds to both these needs.

The de-identification design that we describe in this paper makes clear the importance of developing and implementing both technical and procedural mechanisms to protect patient identity. These mechanisms must be appropriate both in relation to the nature of the data being de-identified and as regards the contexts in which these data will be used. Access to data held within CRIS is tightly monitored, and we undertake significant and regular auditing of CRIS searches. Our approach to de-identification differs from contexts in which de-identified data are widely shared, where the risks of re-identification are arguably greater. CRIS is evaluated regularly to monitor its masking abilities and also to highlight any drawback in de-identification that may occur over time (e.g. new styles of recording PIs, technical glitches, or procedural failures). We have judged the algorithm described here to be optimal for the CRIS dataset: it is a simple module that is highly customizable to deal with changes in data entry, so that when limitations are recognised, large algorithm re-developments are not required and as mentioned above CRIS can also be generalised to other systems if needed.

The algorithm whose development, implementation and evaluation we have presented here shows good precision and recall rates and low probability of combinations of true PIs occurring. We consider this algorithm, alongside the security measures that we have designed to surround its use, to be an example of a simple design with performance comparable to other de-identification algorithms, which advances the development of databases for secondary research with psychiatric case registers.

## Abbreviations

SLaM: South London and Maudsley NHS Foundation Trust; ePJS: Electronic patient journey system; CRIS: Clinical Record interactive Search; PI: Patient identifier(s); NHS: National Health Service; HIPAA: Health Insurance Portability and Accountability Act; SQL: Structured query language; XML: Extensible mark-up language.

## Competing interests

All authors have either substantive or honorary contracts with the South London and Maudsley NHS Foundation Trust which owns the rights to the CRIS system described and stands to gain financially from any wider application.

## Authors’ contributions

FC conceived of the manuscript; AF drafted the manuscript with substantial contributions from all other authors. All authors read and approved the final manuscript.

## Authors’ information

FC is the current chair of the CRIS Oversight Committee and has overseen the development and running of CRIS together with RS, CRIS Academic Lead, MB, CRIS Project Manager and DC, CRIS Developer. AF is the current CRIS Administrator. RGJ is the CRIS Clinical Informatician and has been invaluable in setting up the comparison study with MIST. JT is a CRIS researcher. MS is Head of Information Governance and the Caldicott representative at SLaM. JL is the Research and Development representative jointly with King’s College London and SLaM. RH is a Research Fellow. CKC is the Senior CRIS researcher. AR is a research fellow at the University of Sheffield.

## Pre-publication history

The pre-publication history for this paper can be accessed here:

http://www.biomedcentral.com/1472-6947/13/71/prepub
